# Editorial: Current trends and topics in robotic surgical education in urology

**DOI:** 10.3389/fsurg.2025.1744652

**Published:** 2025-11-27

**Authors:** Nikolaos Liakos, Rudolf Moritz, Martin Janssen, Christian Wagner

**Affiliations:** 1 Department of Urology, Medical Centre of the University of Freiburg, Medical Faculty of the University of Freiburg, Freiburg, Germany; 2 Department of Urology, Marienhospital Herne, Ruhr University, Herne, Germany; 3 Department of Urology, Medical Centre of the University of Münster, Münster, Germany; 4 Department of Urology, Urological Oncology and Robot-Assisted Surgery, St. Antonius Hospital Gronau, Gronau, Germany

**Keywords:** robotics, surgical education, robot-assisted surgery, education, surgical curricula

Robot-assisted surgery has drastically transformed the whole landscape of minimally invasive procedures, offering enhanced precision, optimized ergonomics as well as optimized patient outcomes. As robotic platforms continuously evolve, the educational framework that trains new surgeons changes in character and structure. This editorial explores the emerging trends and needs shaping the future of robot-assisted surgical education.

## From “see one, do one, teach one” to structured curricula

Traditional surgical training relied heavily on the apprenticeship model. In robot-assisted surgery, however, this is not an apposite example of training. The mechanical complexity of current robotic systems, the steep learning curve and the need for reproducible outcomes demand structured competency-based curricula. Structured training programs such as the ERUS Certified Curriculum (ccERUS) exemplify this shift, including simulation, modular assessments and supervised clinical exposure to ensure independent proficiency in procedures like robotic-assisted radical prostatectomy.

## Simulate, simulate and … simulate

Simulation has become a cornerstone of robot-assisted surgical education. High-fidelity virtual reality platforms and dry-lab models allow trainees to develop hand-eye coordination, ambidexterity skills and procedural fluency without any patient risk. Objective metrics enable standardized assessment of both technical and non-technical skills. The integration of augmented reality and AI-driven feedback systems is poised to further personalize as well as accelerate the learning process.

## Non-technical skills and (so desperately needed) team training

Robot-assisted surgery is not a solo endeavor, no “one-man-show”. Effective communication, full situational awareness, and safe decision-making are essential principles in every single operating room. Current educational programs increasingly emphasize non-technical skills, incorporating team-based simulations and further cognitive training. These elements are vital not only for safety but also for shaping not only leadership but also so much needed resilience in surgical theaters.

## Mental health and surgeon wellbeing

A seriously overlooked aspect of surgical education is mental health. The cognitive demands of robot-assisted surgery, combined with the current performance pressure and the irksome ergonomic challenges may and can impact surgeons' well-being. Currently developing training frameworks must address this by promoting reflective feedback, mental support and awareness of possible work-associated burnout. A healthy surgeon is essential for safe surgical practice.

## Looking ahead

The future of robot-assisted surgical education lies in continuous adaptability, outcome-driven refinement of surgical practice and interdisciplinary collaboration. As technology advances, educators must remain agile—integrating innovations while preserving core surgical principles. By fostering a culture of excellence, safety and compassion, we can ensure that robot-assisted surgery continues to benefit patients and strengthen the next generation of surgeons.

